# Characterization of cytoskeletal and junctional proteins expressed by cells cultured from human arachnoid granulation tissue

**DOI:** 10.1186/1743-8454-2-9

**Published:** 2005-10-13

**Authors:** David W Holman, Deborah M Grzybowski, Bhavya C Mehta, Steven E Katz, Martin Lubow

**Affiliations:** 1 Biomedical Engineering Center, The Ohio State University, 260 Bevis Hall, 1080 Carmack Rd, Columbus, OH 43210, USA; 2 Neuroophthalmic Research Group, Department of Ophthalmology, The Ohio State University, Cramblett Hall 5A, 456 W. 10th Ave., Columbus, Ohio 43210, USA; 3 Department of Chemical and Biomolecular Engineering, The Ohio State University, 125A Koffolt Laboratories, 140 W. 19th Ave., Columbus, OH 43210, USA

## Abstract

**Background:**

The arachnoid granulations (AGs) are projections of the arachnoid membrane into the dural venous sinuses. They function, along with the extracranial lymphatics, to circulate the cerebrospinal fluid (CSF) to the systemic venous circulation. Disruption of normal CSF dynamics may result in increased intracranial pressures causing many problems including headaches and visual loss, as in idiopathic intracranial hypertension and hydrocephalus. To study the role of AGs in CSF egress, we have grown cells from human AG tissue *in vitro *and have characterized their expression of those cytoskeletal and junctional proteins that may function in the regulation of CSF outflow.

**Methods:**

Human AG tissue was obtained at autopsy, and explanted to cell culture dishes coated with fibronectin. Typically, cells migrated from the explanted tissue after 7–10 days *in vitro*. Second or third passage cells were seeded onto fibronectin-coated coverslips at confluent densities and grown to confluency for 7–10 days. Arachnoidal cells were tested using immunocytochemical methods for the expression of several common cytoskeletal and junctional proteins. Second and third passage cultures were also labeled with the common endothelial markers CD-31 or VE-cadherin (CD144) and their expression was quantified using flow cytometry analysis.

**Results:**

Confluent cultures of arachnoidal cells expressed the intermediate filament protein vimentin. Cytokeratin intermediate filaments were expressed variably in a subpopulation of cells. The cultures also expressed the junctional proteins connexin43, desmoplakin 1 and 2, E-cadherin, and zonula occludens-1. Flow cytometry analysis indicated that second and third passage cultures failed to express the endothelial cell markers CD31 or VE-cadherin in significant quantities, thereby showing that these cultures did not consist of endothelial cells from the venous sinus wall.

**Conclusion:**

To our knowledge, this is the first report of the *in vitro *culture of arachnoidal cells grown from human AG tissue. We demonstrated that these cells *in vitro *continue to express some of the cytoskeletal and junctional proteins characterized previously in human AG tissue, such as proteins involved in the formation of gap junctions, desmosomes, epithelial specific adherens junctions, as well as tight junctions. These junctional proteins in particular may be important in allowing these arachnoidal cells to regulate CSF outflow.

## Background

Our understanding of cerebrospinal fluid (CSF) egress remains limited regarding fluid movement from the subarachnoid space across the arachnoid granulations (AGs) and into the venous sinuses. The classical view of CSF egress is that arachnoid granulations are herniations of the arachnoid membrane which project into the dural venous sinuses and function to return CSF to the systemic venous circulation [1,2]. In addition, it has long been recognized that there may be a lymphatic component to CSF drainage, recent tracer studies in sheep have suggested that extra-cranial lymphatics might account for as much as 40–48% of CSF outflow [3,4]. Similar results have not yet been demonstrated conclusively in humans, and the relative importance of the two routes at physiologic and non-physiologic intracranial pressures is uncertain. Hence, a study of arachnoidal cell cultures and their proteins will help in the understanding of CSF dynamics.

Impaired CSF circulation can result in increased intracranial pressure, causing hydrocephalus, severe headaches, tinnitus, diplopia, and transient visual obscurations. If left untreated, chronic intracranial pressure can cause intractable headache and compressive optic nerve damage, causing irreversible blindness. To study the role of the arachnoidal cells in CSF outflow and its pathologies, we have developed an *in vitro *model of the CSF outflow pathway across the arachnoid granulations. This model can be applied to physiological as well as pathological conditions of increased intracranial pressure, such as idiopathic intracranial hypertension where it has been suggested that CSF egress is impaired [5] by an increased resistance to outflow at the AGs [6-8]. This *in vitro *model utilizes arachnoidal cells cultured from human AG tissue seeded onto filter membranes as a model for the CSF outflow pathway.

The first step in utilizing this model effectively is to confirm that human arachnoidal cells *in vitro *express some of the cytoskeletal proteins [9,10] and junctional complexes [11] that have been described previously in fixed AG tissue using immunohistochemistry and electron microscopy. In particular, the junctional complexes including gap junctions [11,12], desmosomes [10,11,13-18], epithelial specific cell adhesion molecules (E-cadherin) [19-21], and tight junctions [11,15-18] are important in mediating cell-cell adhesion and communication. These proteins allow the arachnoid cells to form a barrier to CSF egress, regulating the return of CSF to the venous circulation.

The mechanism by which the AG cells facilitate fluid transport is still a topic of debate, though the similarity to the drainage of aqueous humour across the endothelia of Schlemm's canal has been noted by several authors [22,23]. These studies have suggested that AG cells regulate fluid flow by a process of large transcellular vacuoles that originate at the basal membrane of the cell and function as one-way valves [22,24,25]. Ultrastructural studies of human AGs by Yamashima [16,17] have identified large vacuoles and have also suggested that extracellular cisterns between AG cells contribute to the passive transport of CSF, presumably between transiently altered tight junctions.

This paper describes the culture and characterization of the cytoskeletal and junctional proteins expressed by arachnoidal cells grown from human arachnoid granulation explants. Using immunofluorescent microscopy and flow cytometry, we have demonstrated the growth of arachnoidal cells from human AG explants. We have shown that these cells in culture express many of the same cytoskeletal and junctional proteins that have previously been identified in human AG tissue using immunohistochemistry and electron microscopy.

## Methods

### Collection of brain tissue

Human brain tissue was obtained within 24 hours post-mortem from the Ohio State University Regional Autopsy Center. Tissue donors ranged in age from 22 to 88 years old. Brain tissue was collected in accordance with the guidelines and regulations set forth by the Office of Responsible Research Practices Institutional Review Board for human subjects at The Ohio State University (IRB#2002H3018). At autopsy, AGs were collected by resecting the superior sagittal sinus and lateral lacunae. Prior to explantation the tissue was washed 3X in sterile Dulbecco's phosphate buffered saline (D-PBS) (Cellgro Mediatech, Herndon, VA), and then placed into a Petri dish containing fresh culture media. Primary cell culture medium was Dulbecco's Modified Eagle Medium/Ham's F-12 Nutrient medium (50:50 v/v), with L-glutamine, penicillin/streptomycin, amphotericin B (all Cellgro Mediatech), and 10% newborn calf serum (Invitrogen Gibco, Carlsbad, CA).

### Explant procedure and cell culture

The explant procedure was performed under a dissecting microscope. An individual granulation was secured with micro-surgical forceps adjacent to the apical cap cell portion of the granulation. Micro-surgical spring scissors were used to cut just below the forceps as close as possible to the cap of the granulation. Dissecting the apical portion of the granulation ensured that the cap cell cluster was explanted while minimizing the possibility that the fibrous capsule, central core, or underlying arachnoid membrane was explanted as well. The number of explants from each tissue donor depended on the frequency of the AGs on the surface of the brain and in the sinuses. Typical tissue donors generated between 24–48 explants.

The granulation was washed again in fresh culture medium, and explanted into a 24-well culture plate coated with a fibronectin solution of 30 μg human fibronectin (Sigma, St. Louis, MO) per ml M199 culture medium (Cellgro Mediatech). Culture medium was added to each well, and the explants were allowed to remain undisturbed in the incubator for a period of three to four days. The medium was subsequently changed every three to four days as needed. Upon confluency, the cells were washed with D-PBS, and dissociated with 0.05% trypsin EDTA in Hanks buffered saline (Cellgro Mediatech). Trypsinization would typically dissociate the explanted AG tissue as well. At this point sterile forceps were used to remove and discard the explanted tissue. Culture media containing 10% serum was added to the dissociated cells to inhibit the trypsin reaction and the cells were spun down at 1300 RPM. The cell pellet was resuspended and plated to a T-25 cm^2 ^culture flask. In subsequent passages, cells were grown in T-75 cm^2 ^flasks.

### Antibodies

The following monoclonal antibodies were used for immunofluorescence microscopy at the dilutions indicated: mouse anti-human cytokeratin antibody clones AE1/AE3 (1:50, DakoCytomation, Carpinteria, CA), mouse anti-connexin43 antibody (1:100, Zymed, San Francisco, CA), Cy3 conjugated mouse anti-vimentin antibody (1:100, Sigma), mouse anti-human desmoplakin 1&2 antibody (1:40, Chemicon International, Temecula, CA), FITC conjugated mouse anti E-cadherin antibody (1:50, Becton Dickinson, Franklin Lakes, NJ), and FITC conjugated mouse anti ZO-1 antibody (1:50, Zymed). The secondary antibodies used were a fluorescein isothiocyanate (FITC)-conjugated goat anti-mouse IgG_1 _secondary antibody (Sigma) or an Alexa Fluor 555 conjugated donkey anti-mouse IgG1 antibody (Molecular Probes, Eugene, OR) at a 1:50 dilution for 45 minutes at 37°C. All primary and secondary antibodies were diluted in 10% serum from the same species in which the secondary antibody was produced (i.e. goat or donkey serum).

### Immunocytochemistry

Second or third passage cells were seeded onto fibronectin-coated coverslips (Becton Dickinson) and grown to confluency. Cell cultures were tested at 1–1.5 weeks post-confluency for the presence of cytokeratins, vimentin, connexin43, desmoplakins 1&2, E-cadherin, and ZO-1. The cells were washed 3X with sterile D-PBS and fixed with 3.7% paraformaldehyde for 10 minutes, then permeabilized with 0.2% Triton X-100 (Sigma) in PBS at 37°C for 5 minutes. To block non-specific binding of the primary antibody, the cells were incubated for 30 minutes in 10% serum diluted in D-PBS from the same species in which the secondary antibody was produced. Next, the cells were incubated with the primary antibodies at the dilutions indicated above for 60 minutes at 37°C. When appropriate, the cells were washed in D-PBS and incubated with the secondary antibody for 45 minutes. After incubation with the secondary antibody, cells were washed in D-PBS, counterstained with 4',6-diamidino-2-phenylindole (DAPI), and mounted with an antifade reagent (Prolong Gold with DAPI, Molecular Probes) onto glass slides for visualization. The cell cultures were visualized using a Zeiss Axiocam inverted microscope equipped with DAPI, FITC, and Cy3 filter sets. As a negative control, cells grown on a cover slip were stained following the same procedure except the primary antibody was omitted. A lack of staining was interpreted as a high specificity of the primary antibody.

### Labeling with endothelial markers for flow cytometry

Anti-VE-cadherin (CD144) purified IgG_1 _isotype with an anti-IgG_1 _secondary antibody conjugated to phycoerythrin (PE) (Becton Dickinson), and a FITC conjugated anti-CD31 antibody (Becton Dickinson) were used for flow cytometry analysis of approximately 3–5 × 10^6 ^cells. Cell cultures at second or third passage were harvested and spun down, in the same way as for passaging. The cells were resuspended at a concentration of 10^6 ^cells/100 μL. Unlabeled cells were used for control analysis and compensation (10^6 ^cells). The remaining cells were labeled with antibodies VE-cadherin (5 μL /10^6 ^cells) or CD31-FITC (5 μL /10^6 ^cells). The cells were incubated with the primary antibody at 4°C for 25–30 minutes, and then washed with labeling buffer (PBS with 2 mM EDTA, 0.5% bovine serum albumin). For the VE-cadherin experiments cells were then incubated with an anti IgG_1 _PE secondary antibody (10 μL/10^6 ^cells) for 25 minutes. The cells were washed with labeling buffer and finally resuspended in 2% paraformaldehyde.

### Flow cytometry analysis

Analysis was performed on a Becton-Dickinson FACS Calibur equipped with 4 photo multiplier tubes, allowing for 4-color analysis using a 488 nm air-cooled argon and a 633 nm helium-neon laser as excitation wavelengths. VE-cadherin-PE or CD31-FITC expression was assessed within labeled cell populations by comparing their fluorescent expression to unstained control cells. Cells were gated based on their forward and side scattered light (FSC and SSC, respectively) to exclude cellular debris (low SSC and FSC) as well as clumps of cells (large SSC and FSC) that may give erroneous fluorescent readings. Only cells that fell within the defined gate in the light scatter plot were subsequently analyzed for fluorescent expression.

In the subsequent fluorescent analysis, unlabeled cells were examined to determine their intrinsic PE or FITC fluorescence. To determine their intrinsic PE fluorescence, unstained cells were excited with a 488 nm argon laser and their fluorescent emission was detected in the FL2 channel which detects wavelengths from 564–604 nm. Based on their fluorescent emission in the FL2 channel, a gate was established in the PE fluorescence histogram that was considered the intrinsic PE fluorescence by unlabeled arachnoidal cells. In analyzing cells labeled with VE-cadherin-PE, cells were gated based on their light scatter (SSC and FSC) using the same gate as for the unlabeled cells. In the following fluorescent analysis, cells falling within the predefined gate for intrinsic PE fluorescence were considered negative for VE-cadherin-PE expression.

A similar procedure was followed in analyzing cells for CD31-FITC expression except that intrinsic FITC fluorescence was determined in unlabeled arachnoidal cells by exciting cells with a 488 mn argon laser and detecting their fluorescent emission in the FL1 channel which detects fluorescent wavelengths from 515–545 nm.

## Results

### Arachnoidal cell culture

**Figure 1:**
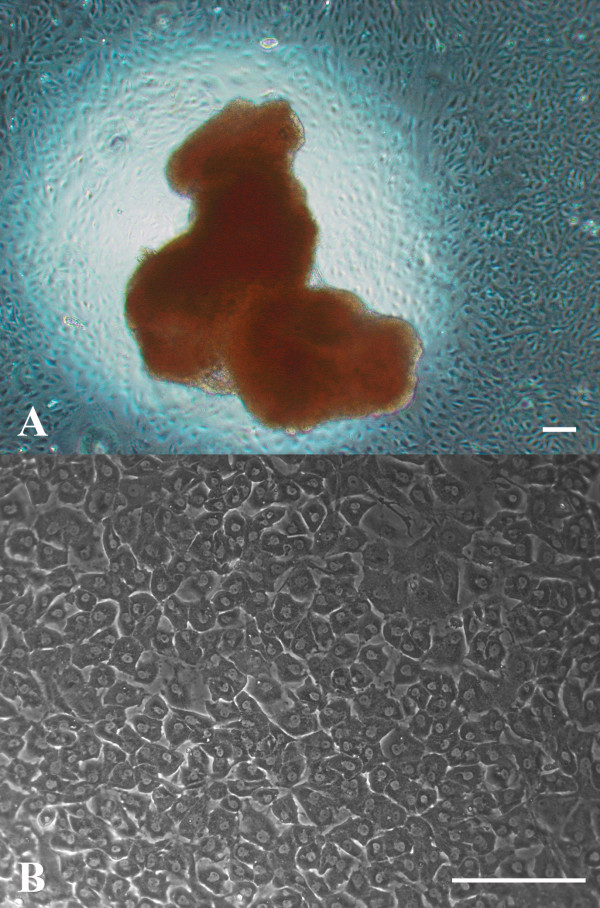
**Human AG explants and arachnoidal cells in culture**. Human AG tissue was obtained at autopsy, usually within 24 hours post-mortem and explanted into cell culture plates coated with fibronectin. A: Cells were seen migrating from the explant within 7–10 days. Bar = 200 μm. B: Arachnoidal cells grown from AG explants in confluent cultures exhibited polygonal cell morphology. Confluent cultures of arachnoidal cells packed in quite densely and displayed a cobblestone-like appearance common to epithelial cells in culture. Bar = 200 μm.

Cell migration from human AG explants was generally seen within 7–10 days (Figure 1A). At confluency, arachnoidal cells exhibited contact inhibition and grew in monolayers with a densely packed polygonal morphology and a cobblestone-like appearance characteristic of epithelial cell types (Figure 1B). Cells could remain in confluent culture for as long as a month and passaged as many as 5–6 times before exhibiting signs of senescence, manifested by enlargement of the cells and the dramatic slowing of cell growth rate.

In limited cases, some cultures became overgrown by fibroblasts, identified as elongated spindle-shaped cells with many processes. Their growth was marked by rapid proliferation that could overgrow the arachnoidal cells in cultures. In instances of fibroblast overgrowth, these cultures were discarded and were not used for immunofluorescent labeling or flow cytometry analysis.

### Immunocytochemical labeling of cytoskeletal and junction proteins

**Figure 2:**
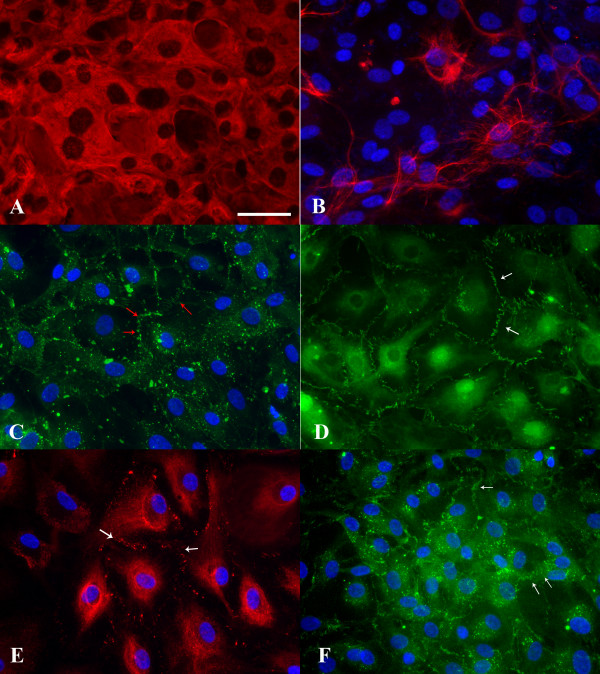
**Immunocytochemical staining of confluent cell cultures from human AG tissue on fibronectin coverslips**. A: Arachnoidal cells in culture were incubated with a Cy3 conjugated anti-vimentin antibody. Cells expressed this intermediate filament protein uniformly throughout the cytoplasm. B: Arachnoidal cells were labeled with a broad spectrum anti-cytokeratin antibody and visualized with a FITC conjugated secondary antibody. Cells expressed the epithelial specific intermediate filament protein cytokeratin in a perinuclear pattern, though the expression of this protein was not uniform. C: Cells cultured from AG tissue were incubated with a connexin43 antibody and then visualized with a FITC conjugated secondary antibody. Immunolocalization of this protein in a punctuate pattern at cell-cell borders (red arrows) indicates that arachnoidal cells are able to form gap junctions in confluent culture. D: Tight junctions in confluent cultures were identified by immunoreactivity to a FITC conjugated ZO-1 antibody. The antibody deposition pattern can be seen at cell-cell borders (white arrow) with overlapping filapodia consisting of short linear structures in parallel. E: Arachnoidal cells in culture were labeled with an anti-desmoplakin antibody and revealed with an Alexa Fluor 555 conjugated secondary antibody. Confluent cultures were able to form desmosomes as evidenced by the punctuate staining along the membranes of adjacent cell borders (white arrows). F: E-cadherin immunoreactivity was demonstrated by incubating cells with a FITC conjugated antibody to E-cadherin. This epithelial specific cell adhesion molecule was expressed at the periphery of the cells, at cell-cell contacts (white arrows) in a pattern similar to that of connexin43 or ZO-1. All immunofluorescent images were taken at the same magnification. Bar= 50 μm.

Cells cultured from human AGs expressed the intermediate filament proteins vimentin and cytokeratin. Second passage cell cultures were immunoreactive to the anti-vimentin antibody (Figure 2A), and cells expressed this protein uniformly throughout the cell cytoplasm. Additionally, a subpopulation of cells in second passage cultures was immunoreactive to the anti-human cytokeratin antibody (AE1/AE3), which recognizes a wide range of human cytokeratins (Moll's designation 1–8, 10, 13–16, 19) [26]. The expression of cytokeratin in the arachnoidal cell cultures varied between experiments. Often, only a subset of stained cells expressed cytokeratins. Immunoreactive cells expressed cytokeratin intermediate filaments in a perinuclear pattern, with long filaments surrounding the nucleus in a basket-like structure (Figure 2B).

Cells cultured from AGs also stained positively for connexin43, with immunofluorescent microscopy showing punctate staining at cell-cell borders (Figure 2C, red arrows). Expression of the ZO-1 protein at the cell membrane between adjacent cells is seen in Figure 2D (white arrows). The antibody deposition pattern can be seen at cell-cell borders with overlapping cellular processes seen as short linear structures in parallel. The expression of desmoplakin 1&2 was similar to that of connexin43, with punctate staining seen along the plasma membrane of two adjoining cells (Figure 2E, white arrows). Finally, Figure 2F shows cells labeled with the FITC-conjugated E-cadherin antibody. This antibody labeled cells at their periphery, at cell-cell contacts (Figure 2D, white arrows), in a pattern similar to the connexin43 or ZO-1 labeled cells.

Cells were also incubated with a FITC conjugated goat anti-mouse IgG_1 _secondary antibody only. The lack of staining in the absence of the primary antibody indicates a high specificity between the primary and secondary antibodies (results not shown). Similar results were obtained for cells incubated with only the Alexa Fluor 555 conjugated donkey anti-mouse IgG1 secondary antibody (results not shown).

Immunocytochemical labeling experiments with antibodies to cytoskeletal and junctional proteins were repeated at least once for cultures grown from the same tissue donor. Immunofluorescent labeling of cytoskeletal and junctional proteins was repeated in at least three additional tissue donors with positive staining seen in all cultures. Images obtained from these experiments were similar to those presented in Figure 2.

### Flow cytometry analysis of endothelial markers

**Figure 3:**
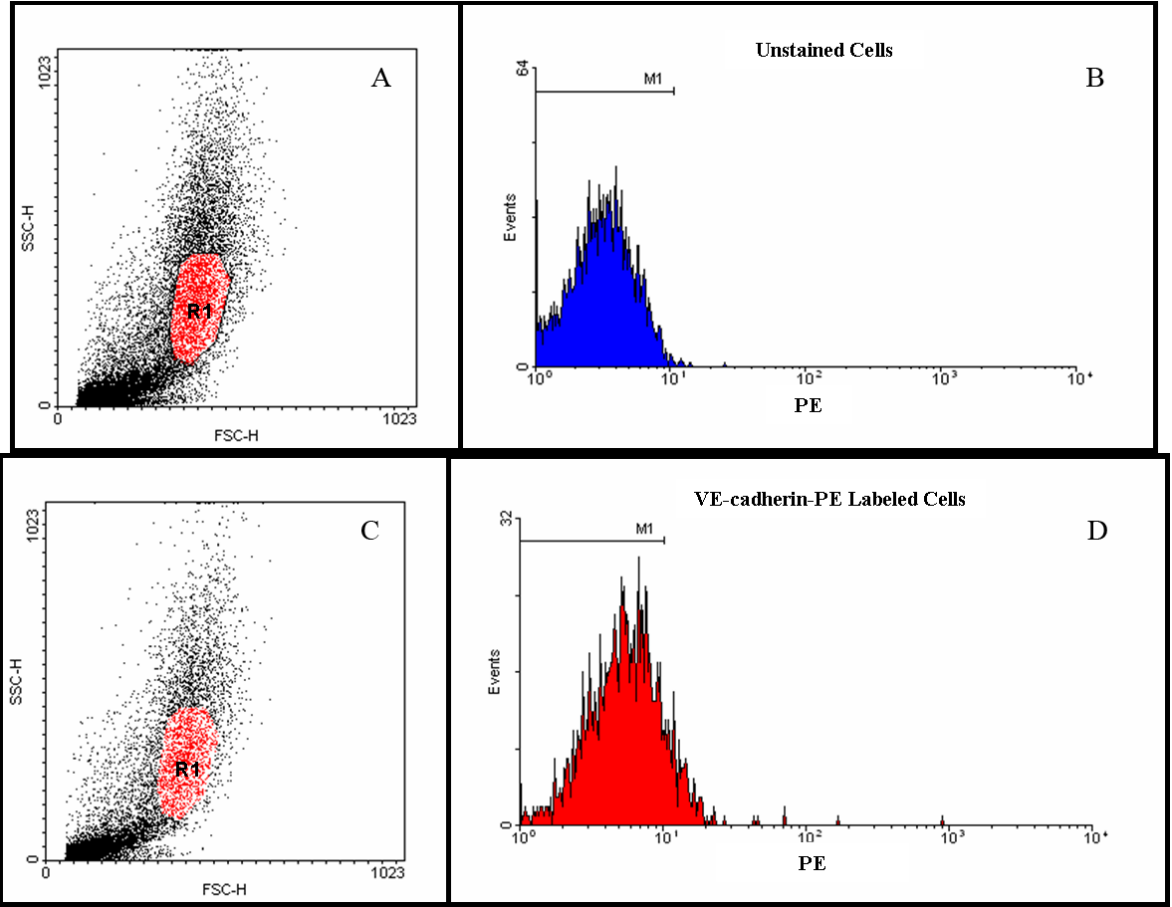
**Flow cytometry analysis of cells cultured from AG tissue for expression of the endothelial marker VE-cadherin-PE**. A: A scatter plot of unlabeled cells, showing a gate, R1, used to define the events subsequently analyzed for fluorescence. SSC and FSC are side scattered light and forward scattered light respectively. B: A histogram was created to determine the autofluorescence of unlabeled cells. The gate M1 is defined as the intrinsic PE fluorescence of unlabeled cells. C: A scatter plots of cells labeled with the endothelial marker VE-cadherin-PE. The position of the gate R1 was unchanged from the control analysis. D: A histogram of the cells labeled with VE-cadherin-PE that fell within the gate R1 and were analyzed for PE fluorescence. Of the 2,601 events that were analyzed, 2,308 (88.7%) fell within the gate, M1, and were considered negative for VE-cadherin expression. The position of the gate, M1, remained unchanged from the control analysis.

Cells cultured from human AGs at second and third passage were labeled with the antibodies to VE-cadherin-PE or CD31-FITC and their expression of these endothelial markers was quantified using flow cytometry. Figure 3A shows the light scatter plot for the unlabeled control cells in the VE-cadherin-PE experiments. The gate R1 (in red) was selected to exclude any cellular debris (low SSC and FSC) and clumps of cells (high SSC and FSC). Only events (cells) falling within this gate were further analyzed for PE fluorescence. Figure 3B shows PE fluorescence histogram used to determine the intrinsic PE fluorescence in unlabeled arachnoidal cell cultures. Gate M1 was created to define the intrinsic PE fluorescence. Of the 4,497 events that fell within R1 and were subsequently analyzed for fluorescence, 4,487 (99.8%) also fell within the gate M1. Figures 3C and 3D show the light scatter plot and PE fluorescence histogram respectively for the cells labeled with the VE-cadherin-PE antibody. The position of the gates R1 and M1 remained unchanged from the control analysis. Of the 2,601 events that fell within R1 and were subsequently analyzed for VE-cadherin-PE expression, 2,308 (88.7%) also fell within gate M1 and were considered negative for VE-cadherin expression.

**Figure 4:**
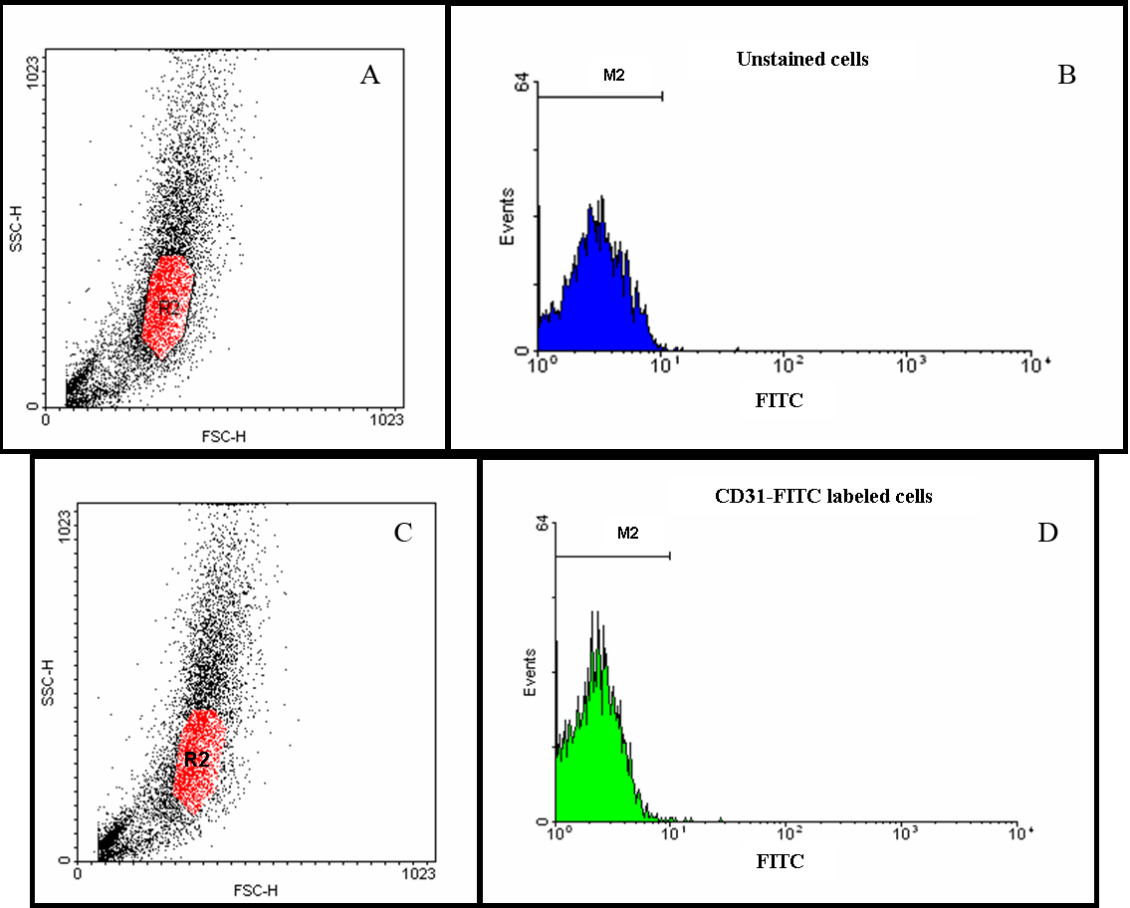
**Flow cytometry analysis of cells cultured from AG tissue for expression of the endothelial marker CD31-FITC**. A: A Scatter plot of unlabeled cells showing a gate, R2, which was created to define events that were subsequently analyzed for fluorescence. SSC and FSC are side scattered and forward scattered light respectively. B: Histogram used to determine the autofluorescence of unlabeled cells, with the gate M2 defined as the intrinsic FITC fluorescence. C: A scatter plot of cells labeled with the CD31-FITC antibody. The position of the gate R2 in the scatter plot was unchanged from the control analysis. D: Histogram of the 3,767 events that fell within R2 and were subsequently analyzed for CD31-FITC expression. Almost all events (99.8%) also fell within the gate M2, indicating that cells cultured from AG tissue did not express the endothelial marker CD31-FITC. The position of gate, M2, did not change from control analysis.

Similar results were obtained for the CD31-FITC labeled cells (Figure 4). The analysis of the unstained control cells is shown in figure 4A and 4B. The light scatter plot in figure 4A also shows the gate, R2 (in red), that was used for subsequent FITC fluorescence analysis. Figure 4B displays the fluorescence histogram that was used to determine the intrinsic FITC fluorescence in unlabeled arachnoidal cell cultures. Of the 3,704 events that fell within the gate R2 and were subsequently analyzed for FITC fluorescence, 3,697 (99.8%) also fell within the gate M2 that was considered the intrinsic FITC fluorescence. Figures 4C and 4D show the light scatter plot and FITC fluorescence histogram respectively for the cells labeled with the CD31-FITC antibody. The position of the gates R2 and M2 remained unchanged from the control analysis. Of the 3,767 events that fell within R2 and were subsequently analyzed for CD31-FITC expression, 3,761 (99.8%) also fell within the gate M2 and were considered negative for CD31 expression. The failure of these cultures to express the endothelial specific proteins CD31 and VE cadherin in significant quantities indicated that cells cultured from human AG explants were not significantly contaminated by endothelia from the venous sinus lumen.

Flow cytometry experiments quantifying the expression of the endothelial markers were repeated with cells cultured from two additional tissue donors. Similar results were obtained to those presented in Figures 3 and 4. Thereafter, it was concluded that endothelial cells were likely not a contaminating cell type and further experiments were not performed

## Discussion

The role of the arachnoid granulations in CSF outflow has been the focus of attention since the works of Weed nearly 90 years ago [1,2]. While the ultrastructure of the arachnoid membrane and granulations has been studied extensively with light and electron microscopy [11,15-18,22,24,25,27-34], functional studies have been few. Because they are located intracranially, it is difficult to observe and measure flow across human AGs *in vivo*. Hence functional studies have been restricted primarily to animal models. Welch and Pollay have described *in vitro *preparations in both monkey and canine [34,35], where excised pieces of dura and arachnoid villi were perfused with colloidal gold, yeast, erythrocytes, and polystyrene microspheres. However, a significant proportion of the preparations developed leaks (7 of 22 in monkeys). In addition, the structure of the arachnoid villi and granulations in primate and canines may not accurately represent the structure of human arachnoid granulations [15,23]. To more closely study the role of arachnoid cells in CSF outflow, we have undertaken efforts to isolate these cells from human AG tissue.

To our best knowledge, this is the first demonstration of primary arachnoidal cell cultures from human AG tissue. Previously leptomeningeal cells have been cultured from the human pia and arachnoid membranes [36-42], but cultures from AG tissue have not been described. This study showed that cells cultured from AG tissue have specific immunological characteristics and can be prepared without contamination of adjacent endothelial cells.

In some limited cases, cultures of arachnoidal cells became overgrown by fibroblasts. Fibroblast contamination was marked by a rapid growth rate that could over grow the arachnoidal cells in culture. However, with the current explant technique, it was possible to consistently grow cultures that were predominantly free of fibroblasts contamination. This was assessed by uniform cobblestone morphology and a homogenous expression of junctional proteins using immunofluorescence.

While care was taken when dissecting the AG tissue, it is possible in some instances that portions of the underlying arachnoid membrane were explanted as well. In such cases, it would be difficult to distinguish cells of the arachnoid membrane from arachnoidal cells from AGs. Differences in protein expression between cells lining the arachnoid granulation and the arachnoid membrane have not been reported and would not be expected, as the AGs are continuous with the arachnoid membrane proper, and are often described as projections of the arachnoid membrane into the dural sinuses. There remain functional differences inferred from differences in ultrastructure between the cells of the arachnoid membrane and those of the arachnoid granulation. The cells lining the arachnoid granulations allow the passage of CSF to the venous sinuses by forming transcellular vacuoles and extracellular cisterns [16-18,22,24,25,43].

The possibility existed that several of the junctional proteins could have been expressed by contaminating endothelial cells from the venous sinus lumen. The flow cytometry analysis, however, demonstrated that arachnoidal cell cultures did not express endothelial markers in significant quantities. Compared to unlabeled control cells, approximately 10% of VE-cadherin-PE labeled cells expressed this marker positively. It was possible that this constituted a small endothelial population within these cultures, though further inspection of this histogram (Figure 3D) did not reveal a second population of PE positive cells. Taken together with the nearly complete lack of CD31 expression (>99%), this strongly suggests that endothelial cells were not present in significant quantities in cultures from AG explants.

To characterize the profile of immunological markers expressed by arachnoidal cells grown from human AG tissue, cultures were labeled with fluorescently conjugated antibodies to several cytoskeletal and junctional proteins. Vimentin intermediate filament expression has been characterized extensively using immunohistochemistry in the cells of the arachnoid membrane, granulations, and meningiomas [9,10,14,44,45], as well as in cultured human leptomeningeal cells [37,39-41]. Cells cultured from AG tissue uniformly expressed vimentin intermediate filaments.

The connexins are found in cells containing gap junctions, providing a pathway for direct intercellular communication for ions, amino acids, and nucleotides. Gap junctions have been identified in human arachnoid membranes and AGs using electron microscopy, freeze fracture, and immunohistochemistry [11,12]. Grafstein *et al*. [39] demonstrated connexin43 expression in cultures of human leptomeningeal cells and showed that these cells could propagate calcium waves suggesting a pathway for intercellular communication. The presence of gap junctions in cell cultured from AGs may provide a pathway for intercellular communication, allowing the regulation of CSF passage.

Desmosomal junctions are typically found in cells of epithelial origin. They function to anchor bundles of intermediate filaments at desmoplakin proteins [46]. While most desmoplakins associate with cytokeratin filaments, exceptions have been found, including the arachnoid cells that anchor vimentin intermediate filaments at these plaques. The presence of desmosomes has been identified with electron microscopy, freeze fracture, and immunohistochemistry [10,11]. Desmosomal junctions are also expressed by *in vitro *cultures of leptomeningeal cells and have been used as a specific marker for these cells [40]. The presence of desmosomes in the *in vitro *cultures from human AGs indicates that these cultures are of arachnoidal origin.

Cadherins are cell adhesion molecules that are largely responsible for calcium dependent cell-cell adhesion. Several types have been described, including N-cadherin in neural and muscle tissue, P-cadherin in placental tissue, E-cadherin found in epithelial cells, and VE-cadherin expressed exclusively in endothelial cells [47,48]. The expression of E-cadherin has been demonstrated in the flash frozen arachnoid membrane, granulations and meningioma tissue using standard immunohistochemistry [19-21]. E-cadherin functions in AG cells to bind the arachnoid cells lining the arachnoid villi and granulations together flexibly and may allow the formation of extracellular cisterns during the bulk outflow of CSF [20]. The *in vitro *expression of E-cadherin by cells cultured from AG tissue demonstrated that these cells were epithelial and could form cell-cell junctions that may be necessary to regulate the passage of fluid.

The zonula occludens (ZO proteins) are peripheral membrane proteins that associate with other proteins in tight junctions where they serve to anchor the actin cytoskeleton. In epithelial cells, these junctions maintain polarity and regulate the paracellular passage of ions, macromolecules, and water. While tight junctions have been recognized in the arachnoid cells lining the AGs [15-17], these junctions have not been described in cells cultured from the leptomeninges. Demonstrating the presence of tight junctions in cells cultured from AG tissue cultures, suggest that these cells have the potential to form a barrier to fluid flow.

The expression of cytokeratin filaments in the arachnoid membrane and granulations has been the subject of debate. The association of intermediate filaments with desmosomal junctions in meningiomas, arachnoid membrane, and arachnoid granulations was first reported by Kartenbeck *et al*. and Schwecheimer *et al*. [10,14], who demonstrated that desmosomal plaques anchored vimentin filaments, while cytokeratin immunoreactivity within these tissues was not found. Subsequent immunohistochemical studies have also failed to find cytokeratin expression in the meninges and AGs [44,45,49]. However, cytokeratin positive cells within the arachnoid have been recognized in limited cases [50,51]. Several published reports of cultured leptomeningeal cells show that these cells express cytokeratin uniformly [38,40,41]. Of note are studies by Frank *et al*. [38] and Murphy *et al*. [40] focusing on cultured leptomeningeal cells grown from human arachnoid membranes.

The variability in cytokeratin expression may be related to the embryological origin of the meninges. The development of the meninges has been described in detail by O'Rahilly and Müller [52], who suggest that there are several possible sources for the human cranial meninges, including the parachordal mesoderm, mesectoderm, and other neuroectodermal elements. Embryological contribution from mesodermal as well as neuroectodermal elements may explain the later mesenchymal and epithelial properties of the adult meninges. Arachnoid cells appear to express both epithelial and mesenchymal properties [36,44,45,49]; mesenchymal by their ability to express vimentin intermediate filaments and synthesize basement membrane proteins (collagen type I and IV, fibronectin, laminin) [37], and epithelial through the formation of cell-cell junctions [11].

Since cytokeratin expression has not been found consistently in human arachnoid membrane and granulation tissue, it seems anomalous that cells cultured from these tissues should express cytokeratins uniformly. In identifying arachnoidal cells within these cultures, the formation of cell-cell junctions may be more relevant to recapitulate these cells' *in vivo *function, regulating the outflow of CSF.

Future studies will focus on characterizing these cells' ability to form an occluding barrier to fluid flow using a perfusion chamber and hydrostatic pressure control. Cells are perfused and fixed under pressure, and the cellular ultrastructure is examined using electron microscopy. These studies will also try to determine whether arachnoidal cells *in vitro *can mimic the one-directional flow of CSF *in vivo*.

## Conclusion

These results show what we believe to be the first demonstration and characterization of arachnoidal cells cultured from human AG tissue. We have demonstrated the *in vitro *expression of several important cytoskeletal and junctional proteins previously identified in AG tissue by immunohistochemistry and electron microscopy. The expression of these junctional proteins is an important first step in demonstrating that arachnoidal cells grown from human AG tissue *in vitro *can exhibit some of the barrier properties previously recognized in intact AG tissue and may allow these cells to regulate CSF outflow.

## List of Abbreviations

AG: Arachnoid granulation, CSF: cerebrospinal fluid, DAPI: 4',6-diamidino-2-phenylindole, D-PBS: Dulbecco's phosphate buffered saline, FITC: fluorescein isothiocyanate, FSC: forward scatter, PE: phycoerythrin, SSC: side scatter

## Competing interests

The author(s) declare that they have no competing interests.

## Authors' contributions

DH: collected and dissected brain tissue, performed immunocytochemical staining, with BM prepared and analyzed cells with flow cytometry, and drafted and revised the manuscript.

DG: conceived of the study, developed the initial explant procedures, participated in the study design and coordination, and helped to draft and revise the manuscript.

BM: with DH prepared and analyzed cells with flow cytometry.

SEK: participated in the design and coordination of the study, helped to revise the manuscript.

ML: participated in the design and coordination of the study, helped to revise the manuscript.

All authors read and approved the final manuscript.
